# Treatment Outcomes in Vertical Shear Pelvic Fractures: A Comparative Study

**DOI:** 10.7759/cureus.65500

**Published:** 2024-07-27

**Authors:** Ahmed Ashour, Ehab Alieldin, Ahmed Ismail, Ahmed T Ashour, Ahmed Abouelnaga, Ahmed M Attia, Mahmoud Salama

**Affiliations:** 1 Trauma and Orthopaedics, Queen Elizabeth Hospital Birmingham, Birmingham, GBR; 2 Trauma and Orthopaedics, The Royal London Hospital, London, GBR; 3 Orthopaedics, Bradford Royal Infirmary, Bradford, GBR; 4 Orthopaedics and Trauma, El Hadara University Hospital, Alexandria, EGY; 5 Trauma and Orthopaedics, Manchester Royal Infirmary, Manchester, GBR; 6 Trauma and Orthopaedics, Barts Health NHS Trust, London, GBR; 7 Trauma and Orthopaedics, University Hospitals of Alexandria, Alexandria, EGY

**Keywords:** lumbosacral, hemipelvis, lumbopelvic fixation, iliosacral screw, vertical shear fractures pelvis

## Abstract

Background

Vertical shear (VS) pelvic ring injuries present a unique challenge due to their inherent vertical and rotational instability and the risk of massive bleeding. VS injuries may result from either bony or ligamentous injury. The goal in the treatment of VS fractures of the pelvis is to achieve and maintain an accurate reduction of the displaced hemipelvis.

Aim of the study

This study aimed to compare the results of the treatment of VS fractures pelvis by using iliosacral (IS) screws versus lumbopelvic fixation (LPF).

Methodology

This retrospective study was carried out on 40 patients with VS fracture pelvis injuries at El Hadara University Hospital, Alexandria, Egypt, from January 2020 to December 2020. Twenty of them were treated by an IS screw, and the other 20 were treated by LPF. Then, both groups were followed up for six months with regard to union rate, metal failure, and clinical outcomes.

Results

The EQ-5D showed a significant improvement in LPF more than the IS screw group in the five items of the score. Moreover, the total EQ-5D index showed a significant increase in the LPF group more than the IS screw group (p < 0.05). The incidence of neurological complication was found in four cases in the IS screw group, while no cases were found in the LPF group. The infection was found in six patients in the IS screw group and only three cases in the LPF group. The malunion was found in two cases in the IS screw group and no cases in the LPF group. The neurological change and the incidence of infection were significantly higher in the IS screw group than in the LPF group (p < 0.05).

Conclusion

Our results demonstrate reliable maintenance of reduction and acceptable complication rates with a minimally invasive LPF for VS fractured pelvis. The benefits of minimally invasive LPF may be offset by increased elective reoperations for the removal of instrumentation.

## Introduction

Vertical shear (VS) pelvic ring injuries present a unique challenge due to their inherent vertical and rotational instability and risk of massive bleeding [1]. These injuries, resulting from either bony or ligamentous trauma, require precise management strategies to achieve and maintain accurate reduction of the displaced hemipelvis [2].

The short-segmented posterior internal fixator is widely regarded as the gold standard for initial reduction and stabilization, but without additional treatment of the anterior column, significant correction loss or implant failure may occur, particularly in cases involving VS patterns, which as a result may decrease early progressive weight-bearing. These injuries are associated with a spectrum of complications that can adversely affect patient outcomes, including chronic low back pain, limb length discrepancy, gait abnormalities, and functional impairments, such as difficulty with sitting, sexual dysfunction, and bowel or bladder incontinence [3-5]. Clinical studies focusing exclusively on VS injuries are limited due to their relative rarity, often necessitating their inclusion with other vertically unstable pelvic ring injuries for comprehensive analysis [6].

Iliosacral (IS) screws, which traverse one sacroiliac joint and terminate in the sacrum, are commonly used for posterior pelvic ring fixation. However, fixation failure and nonunion remain concerns, especially in the context of vertical sacral fractures. Transition screws (TS) that extend across both sacroiliac joints have gained popularity due to their potentially enhanced stability, although biomechanical studies have shown mixed results regarding their superiority over IS screws [7-9]. Lumbopelvic fixation (LPF) represents an adjunctive technique used in cases of significantly unstable fractures, offering additional stabilization, particularly in the presence of spinopelvic instability. LPF may play a critical role in managing complex sacral fractures with anterior instability, potentially reducing complications associated with correction loss and implant failure [10,11].

In summary, the treatment of VS pelvic fractures requires a tailored approach that balances the need for anatomical reduction and stable fixation to minimize complications and optimize functional outcomes. This study aims to compare the efficacy and outcomes of IS screws versus LPF in the management of these challenging injuries.

## Materials and methods

Study design and participants

This retrospective study was carried out on 40 patients with VS fracture pelvis injuries at El Hadara University Hospital, Alexandria, Egypt, from January 2020 to December 2020. Twenty of them had IS screws, and the other 20 were treated by LPF, and then both groups were followed up for six months as regards the union rate, metal failure, and clinical outcomes. We included patients aged 18 and older, diagnosed with VS fracture of the pelvis, with complete six-month follow-up data. On the other hand, the exclusion criteria were patients with incomplete data, other types of pelvic fractures, significant additional injuries, prior pelvic surgeries, non-surgical management, and under 18-year-old patients.

Data collection and evaluation

The SI screw method was performed using a standardized percutaneous technique in the prone position. The decision to use single or dual screws and their length were taken preoperatively by the senior author depending on the screw purchase and fracture morphology, non-comminuted longitudinal fractures, acceptable closed reduction with a residual displacement <1 cm, absence of neurodeficit or lumbosacral dysmorphism, and absence of high transverse fracture were indicated. On the other hand, LPF was performed using a paraspinal approach for unilateral injuries with normal neurology and a midline approach for bilateral injuries or those with neurological deficits using an L4/L5 pedicle screw (extension to L4 in the L5 pedicle fracture/L4-5 pre-existing instability), iliac screw, and connecting rod. Neurological deficit, comminuted sacral fracture, lumbosacral dysmorphism, an extension of fracture into the L5-S1 facet, high transverse fractures, and failure of closed reduction were indicated.

Demographic and injury information was collected from the trauma registry. Immediate postoperative anteroposterior, inlet, and outlet radiographs and similar follow-up views from a minimum of 12 months post-injury were examined. The position, length, and number of IS screws and any evidence of metalwork failure (e.g., bending or breakage) were recorded. The observers measured vertical displacement on each radiograph by constructing horizontal reference lines through bony landmarks, and the displacement of the innominate bone in a posterior or superior direction was taken as positive.

The main outcome measure in this study was defined as failure, specifically characterized by a minimum of 1 cm of combined vertical displacement of the posterior pelvis compared to the immediate postoperative position. This criterion was chosen to assess the effectiveness of the treatment approaches in maintaining anatomical reduction and stability over time [12,13].

The EQ-5D questionnaire was utilized to measure health-related quality of life across five dimensions: mobility, self-care, usual activities, pain or discomfort, and anxiety or depression. Each dimension was scored based on three levels of severity (no problem, moderate problem, and severe problem), providing a detailed assessment of patient-reported health status. In addition, the EQ VAS allowed patients to self-rate their overall health on a scale from 0 to 100, with higher scores indicating better perceived health. The EQ-5D index score, derived from a scoring algorithm, provided a summary measure ranging from 1 (full health) to 0 (death), offering insights into the relative health status compared to perfect health [14,15]. Permission to use the EQ-5D questionnaire was obtained from the EuroQol Research Foundation. The necessary authorization was granted, and all conditions set forth by the EuroQol Research Foundation were adhered to throughout the study.

These outcome measures and assessments were critical in evaluating both the clinical efficacy and patient-reported outcomes of the different treatment modalities (iliac screw vs. LPF) for VS pelvic fractures. They aimed to provide comprehensive data on treatment success rates, functional recovery, and quality of life improvements, guiding future treatment protocols and optimizing patient care strategies.

Statistical analysis

Data collection was meticulously carried out and subsequently entered into a personal computer for analysis. For statistical analysis, we utilized the IBM SPSS Statistics for Windows, Version 24.0 (released 2016, IBM Corp., Armonk, NY), which is a comprehensive tool for data management and analysis. For the analysis of categorical parameters, we calculated the arithmetic mean and standard deviation to summarize the central tendency and dispersion of the data. To assess the relationships between categorical variables, the chi-square test was employed. This test is particularly useful for determining if there is a significant association between two categorical variables. For numerical data, the independent samples t-test was used to compare the means between the two groups. The t-test is a robust statistical method for determining if there are statistically significant differences between the means of two independent groups, providing insights into the effectiveness of the different treatment approaches.

## Results

This study was carried out on 40 patients with VS fracture pelvis; 20 of them used IS screws and the other 20 used LPF. The basic demographic and clinical data showed that the mean age in the IS screw group was 35.4 ± 12.6 years, while in the LPF group, it was 33.4 ± 11.4. The male/female ratio in the IS screw group was 11:9, and in the LPF group, it was 14:6. There was no significant difference between the two studied groups regarding age and sex. The major mode of injury in the two groups was road traffic accident (RTA), followed by falling. Moreover, there was no significant difference between the two groups regarding the mode of injury. The time lapse and follow-up period in the two groups were matched without a significant difference. The major classification in the two groups was B2, and the majority of the patients were undisplaced (Table 1).

**Table 1:** Basic demographic and clinical data of the two studied groups. ^*a* ^corresponds to the values originally denoted by the t-test. *^b^* corresponds to the values originally denoted by the chi-square test.

Parameter	Iliosacral screw (n = 20)	Lumbopelvic fixation (n = 20)	Statistical test result	P-value
Age (years)	35.4 ± 12.6	33.4 ± 11.4	0.616*^a^*	0.542
Sex	‐	‐	0.671*^b^*	0.412
Male	11 (55.0%)	14 (70.0%)	‐	‐
Female	9 (45.0%)	6 (30.0%)	‐	‐
Mode of injury		‐	0.322*^b^*	0.57
RTA	14 (70.0%)	15 (75.0%)	‐	‐
Falling	6 (30.0%)	5 (25.0%)	‐	‐
Time lapse (days)	3.8 ± 2.3	4.75 ± 4.0	-1.452*^a^*	0.154
Follow-up period (months)	26.1 ± 17.0	32.0 ± 16.5	-1.065*^a^*	0.295
Classification		‐	2.187*^b^*	0.703
B1	0 (0.0%)	1 (5.0%)	‐	‐
B2	12 (60.0%)	10 (50.0%)	‐	‐
B3	6 (30.0%)	5 (25.0%)	‐	‐
C1	1 (5.0%)	3 (15.0%)	‐	‐
C2	1 (5.0%)	1 (5.0%)	‐	‐
C3	0 (0.0%)	0 (0.0%)	‐	‐
Displacement		‐	0.215*^b^*	0.643
Un-displaced	16 (80.0%)	15 (75.0%)	‐	‐
Displaced	4 (20.0%)	5 (25.0%)	‐	‐

Between the two studied groups, the EQ-5D showed a significant improvement in the LPF group more than IS screw group in the five items of the score. Moreover, the total EQ-5D index showed a significant increase in the LPF group more than the IS screw group (p < 0.05) (Table 2).

**Table 2:** Comparison between the two studied groups regarding EQ-5D ^a^ corresponds to the values originally denoted by the t-test. ^b^ corresponds to the values originally denoted by the chi-square test. * means statistically significant at p ≤ 0.05.

Parameter	Iliosacral screw (n = 20)	Lumbopelvic fixation (n = 20)	Statistical test result	P-value
Mobility	‐	‐	‐	‐
Normal	10 (50.0%)	14 (70.0%)	4.60*^b^*	0.032*
Moderate problems	4 (20.0%)	4 (20.0%)	0.114*^b^*	0.735
Severe problems	6 (30.0%)	2 (10.0%)	2.39*^b^*	0.123
Self-care	‐	‐	‐	‐
Normal	9 (45.0%)	15 (75.0%)	7.80*^b^*	0.0205*
Moderate problems	5 (25.0%)	4 (20.0%)	0.320*^b^*	0.567
Severe problems	6 (30.0%)	1 (5.0%)	4.17*^b^*	0.041*
Usual activities		‐	‐	‐
Normal	7 (35.0%)	16 (80.0%)	11.01*^b^*	0.001*
Moderate problems	10 (50.0%)	4 (20.0%)	6.33*^b^*	0.012*
Severe problems	3 (15.0%)	0 (0.0%)	2.52*^b^*	0.112
Pain	‐	‐	‐	‐
Normal	8 (40.0%)	15 (75.0%)	4.17*^b^*	0.041*
Moderate problems	7 (35.0%)	3 (15.0%)	1.28*^b^*	0.258
Severe problems	5 (25.0%)	2 (10.0%)	3.20*^b^*	0.074
Anxiety/depression		‐	‐	‐
Normal	11 (55.0%)	16 (80.0%)	4.33*^b^*	0.038*
Moderate problems	6 (30.0%)	3 (15.0%)	1.55*^b^*	0.214
Severe problems	3 (15.0%)	1 (5.0%)	0.74*^b^*	0.389
EQ-5D Index	0.75 ± 0.198	0.832 ± 0.211	-2.145*^a^*	0.036*

The incidence of neurological complication was found in four cases in the IS screw group, while no cases were found in the LPF group. The infection was found in six patients in the IS screw group and only three cases in the LPF group. The malunion was found in two cases in the IS screw group and no cases in the LPF group. The neurological change and incidence of infection were significantly higher in the IS screw group than in the LPF group (p < 0.05) (Table 3).

**Table 3:** Comparison between the two studied groups regarding postoperative complication *^b^* corresponds to the values originally denoted by the chi-square test. * means statistically significant at p ≤ 0.05.

Parameter	Iliosacral screw (n = 20)	Lumbopelvic fixation (n = 20)	Statistical test result	P-value
Neurological changes		‐	‐	‐
Yes	4 (20.0%)	0 (0.0%)	4.17^b^	0.042*
No	16 (80.0%)	20 (100.0%)	1.42^b^	0.158
Infection	‐	‐	‐	‐
Yes	6 (30.0%)	3 (15.0%)	3.90^b^	0.048*
No	14 (70.0%)	17 (85.0%)	1.60^b^	0.207
Failure (malunion)		‐	‐	‐
Yes	2 (10.0%)	0 (0.0%)	2.65^b^	0.104
No	18 (90.0%)	20 (100.0%)	1.07^b^	0.302

## Discussion

The objective of this study was to compare the results of the treatment for VS pelvic fractures using IS screws versus LPF. Our findings revealed several important insights into the efficacy and outcomes of these two treatment approaches.

In comparing our results with previous studies, we found that the use of IS screws showed comparable fracture reduction rates to LPF [7-9]. This is consistent with the findings of Wenning et al. (2021) who also reported successful fracture reduction, reduced operative time, reduced length of hospital stay, and lower infection rates with IS screws. However, in terms of functional outcomes, LPF appeared to offer superior results [10].

The observed differences in functional outcomes between the two treatment approaches may be attributed to the biomechanical advantages of LPF. By providing rigid stabilization and load-sharing across the entire pelvic ring, LPF may offer better stability and improved fracture healing compared to IS screws alone [11,16]. This could result in enhanced functional outcomes and reduced long-term complications, such as chronic pain or sacroiliac joint dysfunction.

Based on our findings, we recommend considering LPF as the primary treatment approach for VS pelvic fractures, particularly in cases where functional outcomes are a priority. However, the decision between IS screws and LPF should be individualized, taking into account patient factors, fracture characteristics, and surgeon expertise.

Future research should aim to investigate the long-term outcomes and cost-effectiveness of these treatment approaches. In addition, studies exploring the role of minimally invasive techniques or patient-specific biomechanical simulations could provide valuable insights into optimizing the treatment of VS pelvic fractures [9].

It is important to acknowledge the limitations of our study. First, the sample size was relatively small, which may have limited the generalizability of our findings. Second, the follow-up duration was limited to one year, and longer-term outcomes were not assessed. Further studies with larger sample sizes and longer follow-up durations are needed to validate our findings and provide a more comprehensive understanding of the treatment outcomes for VS pelvic fractures.

## Conclusions

Our findings indicate that minimally invasive LPF for VS fractures of the pelvis provides consistent and stable reduction with a satisfactory rate of complications. This technique proves to be effective in maintaining alignment and offers a less invasive option compared to traditional methods. However, the advantages of this minimally invasive approach come with a trade-off; there is a higher likelihood of patients requiring elective reoperations to remove the implanted hardware. Despite this, the overall benefits, including reduced surgical trauma and quicker recovery times, suggest that minimally invasive LPF remains a viable and beneficial treatment option for these complex fractures.
